# Occupational hazards and the development of lung cancer: an analysis of exposures and their implications for worker health

**DOI:** 10.47626/1679-4435-2025-1417

**Published:** 2025-09-14

**Authors:** Matheus de Sousa Alves, Luan Santana Santos de Carvalho, Luciana Tolstenko Nogueira, Sissi Andrade Sá Furtado

**Affiliations:** 1 Departamento de Medicina, Universidade Estadual do Piauí, Teresina, PI, Brazil.; 2 Departamento de Odontologia, Universidade Estadual do Piauí, Teresina, PI, Brazil.

**Keywords:** lung neoplasms, occupational exposure, occupational health, carcinogens, environmental, disease prevention, neoplasias pulmonares, exposição ocupacional, saúde ocupacional, carcinógenos ambientais, prevenção de doenças

## Abstract

Lung cancer is one of the leading causes of death worldwide and is associated with occupational exposure to carcinogenic agents such as asbestos, silica, welding fumes, hydrocarbons, and heavy metals. This study analyzed the occupational risks related to lung cancer and their implications for workers’ health. It is an integrative literature review, with searches conducted in SciELO, LILACS, and PubMed databases as well as institutional publications. A total of 13 studies were selected, including systematic reviews, meta-analyses, and observational studies, providing a comprehensive view of the impact of prolonged exposure to carcinogenic substances in sectors such as construction, mining, and the chemical industry. The results show an association between occupational exposure and an increased incidence of lung cancer among workers. Weaknesses in inspection and in the implementation of preventive measures exacerbate this scenario. Studies highlight the need for stricter public policies, stronger regulations, and the expansion of early screening programs for exposed workers. Replacing harmful substances, combined with effective preventive measures, can significantly reduce cases of occupational lung cancer.

## Abstract (pt)

O câncer de pulmão é uma das principais causas de morte no mundo, sendo associado à exposição ocupacional a agentes carcinogênicos, como amianto, sílica, fumaça de solda, hidrocarbonetos e metais pesados. Este estudo analisou os riscos ocupacionais relacionados ao câncer de pulmão e suas implicações para a saúde do trabalhador. Trata-se de uma revisão integrativa da literatura, com buscas realizadas nas bases SciELO, LILACS e PubMed, além de publicações institucionais. Foram selecionados 13 estudos que abrangem revisões sistemáticas, metanálises e estudos observacionais, o que proporcionou uma visão ampla sobre o impacto da exposição prolongada a substâncias cancerígenas em setores como construção civil, mineração e indústria química. Os resultados evidenciam uma associação entre a exposição ocupacional e o aumento da incidência de câncer de pulmão em trabalhadores. A precariedade na fiscalização e na implementação de medidas preventivas agrava esse cenário. Estudos destacam a necessidade de políticas públicas mais rigorosas, reforço na regulamentação e ampliação de programas de rastreamento precoce para trabalhadores expostos. Conclui-se que a substituição de substâncias nocivas, aliada a medidas de prevenção eficazes, pode reduzir significativamente os casos de câncer de pulmão ocupacional.

## Introduction

Lung cancer is defined by the American Cancer Society^1^ as a malignant neoplasm that develops in lung tissue, usually in the cells lining the airways. It is estimated to be responsible for 1.8 million deaths annually, accounting for 18% of all cancer-related deaths. In Brazil, lung cancer ranks as the second most common cancer among men and the fourth among women.^2^ This disease represents approximately 17% of all malignant neoplasms diagnosed each year in the country.^3^

The condition is classified into two main categories: non-small cell lung cancer (NSCLC) and small cell lung cancer (SCLC). NSCLC is the most prevalent type and includes subtypes such as adenocarcinoma, squamous cell carcinoma, and large cell carcinoma. SCLC, although less common, tends to be more aggressive and is often associated with smoking. Risk factors include exposure to tobacco smoke, air pollution, radon, and other carcinogenic agents, with most cases occurring in individuals with a significant history of smoking.^4^

The incidence of lung cancer increased dramatically from the late 19th century onward due to the popularization of cigarette smoking, now recognized as its leading cause.^5^ However, the risk is even greater when smoking is combined with exposure to chemical or physical agents in the workplace, as highlighted in the Atlas of Work-Related Cancer in Brazil.^6^ This interaction produces a synergistic effect that not only adds but multiplies the chances of developing the disease, potentially increasing the magnitude of risk and shortening the latency period for tumor onset, as seen in the classic association between smoking and exposure to asbestos.^6^

The relationship between lung cancer and occupational factors is widely discussed, as continued exposure to carcinogenic substances — especially chemical agents such as asbestos, benzene, and volatile organic compounds — has been linked to higher incidence of lung cancer in heavily exposed populations.^7^ In Brazil, economic sectors such as construction, mining, and the chemical industry are of particular concern, as workers in these fields often handle these chemical agents without adequate protection, increasing their risk of developing the disease.^8^

Workers exposed to mineral residues and chemical products face an increased risk of developing lung cancer, emphasizing the need for occupational health surveillance and appropriate workplace interventions.^9^

Furthermore, inadequate regulation and insufficient enforcement of protective and safety measures in the workplace contribute to the persistence of this public health problem. Studies indicate that public policies focused on raising awareness about occupational risks and promoting safe practices are essential for preventing lung cancer among exposed workers.^10^ This proposed integrative literature review aims to compile and analyze the available evidence on occupational lung cancer in Brazil.

## Methods

This study consists of an integrative literature review, an approach that enables the synthesis of knowledge through the analysis of different types of previously published scientific studies. This methodology was chosen for its ability to integrate findings from observational research and systematic reviews, allowing for a comprehensive understanding of occupational lung cancer in Brazil.

The search for scientific articles was conducted in SciELO, LILACS, and PubMed databases. In addition to these sources, institutional publications from organizations such as the Brazilian Ministry of Health and the World Health Organization (WHO) were consulted to complement the information. Descriptors were selected from the Descriptors in Health Sciences (DeCS) controlled vocabulary and adapted to fit the research scope. The main terms included: lung cancer, lung neoplasms, occupational exposure, workers, carcinogenic agents, industry, occupational health, and Brazil, along with their equivalents in English and Spanish.

The search strategy was structured based on the PICO (Patient/Problem, Intervention, Comparison, and Outcome) framework. The problem investigated was lung cancer associated with occupational exposure in Brazil; the intervention was the analysis of working conditions and exposure to carcinogenic agents in different industrial sectors; no comparison was specified due to the focus on observational studies; and the expected outcome was the identification of risk factors and trends related to the occupational impact on disease incidence.

Inclusion criteria were studies addressing the relationship between lung cancer and occupational exposure in Brazil; published in Portuguese, English, or Spanish; available in full text; peer-reviewed; and focused on publications from the last 10 years (2014-2024) to ensure up-to-date information. Exclusion criteria were articles that did not specifically address the relationship between occupational exposure and lung cancer, duplicates across databases, studies unavailable in full text, and experimental studies in animal or in vitro models without direct application to human occupational health.

The systematic search was conducted between September and December 2024, with an update in February 2025. Article selection occurred in three stages: first, an initial screening by title and abstract to eliminate irrelevant studies; then, full-text reading of the selected articles to confirm they met the inclusion criteria; and finally, extraction and organization of data into categories, highlighting risk factors, most affected industrial sectors, and preventive measures. To ensure the reliability of the analysis, two researchers performed the selection independently, and in cases of disagreement, a third reviewer was consulted.

**Figure 1.**
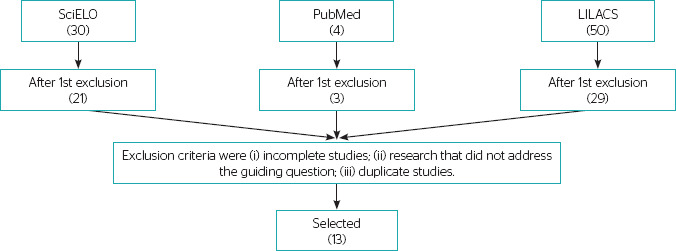
Steps for selecting articles in the described databases, Teresina, Piauí, 2024 (n = 13).

After applying the inclusion and exclusion criteria, 53 articles were selected, with 21 from PubMed, 3 from SciELO, and 29 from LILACS (Figure 1). After further analysis, 13 articles were included in this review. Table 1 presents the search structure and the results obtained after duplicate removal.

**Table 1:** Results of the systematic search in the databases

Database	Search terms	Results found (after removal of duplicates)	Articles included
PubMed	(“lung cancer” OR “pulmonary cancer”) AND (“occupational exposure” OR “workers” OR “industrial sector”) AND (“Brazil” OR “Brazilian”)	30	21
SciELO	(“câncer de pulmão” OR “câncer pulmonar”) AND (“exposição ocupacional” OR “trabalhadores” OR “setor industrial”) AND (“Brasil” OR “brasileiro”)	4	3
LILACS	(“lung cancer” OR “pulmonary cancer”) AND (“occupational exposure” OR “workers” OR “industrial sector”) AND (“Brazil” OR “Brazilian”) AND (“câncer de pulmão” OR “câncer pulmonar”) AND (“exposição ocupacional” OR “trabalhadores” OR “setor industrial”) AND (“Brasil” OR “brasileiro”) AND (“cáncer de pulmón” OR “cáncer pulmonar”) AND (“exposición ocupacional” OR “trabajadores” OR “sector industrial”) AND (“Brasil” OR “brasileño”)	50	29

LILACS = Latin American and Caribbean Literature on Health Sciences; SciELO = Scientific Electronic Library Online.

After the rigorous application of the inclusion and exclusion criteria — which prioritized studies published between 2014 and 2024 and eliminated duplicate, irrelevant, or out-of-scope articles — a final corpus of 13 articles was obtained for analysis from the 53 initially selected across the databases.

## Results

The studies analyzed were published between 2016 and 2024, mostly in Brazilian and international scientific journals such as Revista Gaúcha de Enfermagem, Revista Brasileira de Medicina do Trabalho, International Journal of Environmental Research and Public Health, and Jornal Brasileiro de Pneumologia. Searches were conducted by various authors, including both Brazilian and international specialists, reflecting a global concern regarding the impact of occupational exposure on the development of lung diseases and cancer.

The methodologies employed included systematic reviews, meta-analyses, case-control studies, and biomarker analyses, ensuring a broad and detailed scope. Most studies emphasized the association between occupational exposure to carcinogenic substances — such as asbestos, silica, welding fumes, hydrocarbons, and heavy metals — and the development of lung cancer and other pulmonary diseases.

Table 2 presents the main risk factors and the key findings from the analyzed studies.

**Table 2:** Selected bibliographic sources, classified by authorship (year), study type, risk factors, and main findings related to occupational cancer, 2016-2024 (n = 13)

	Title	Year	Type of study	Lead author	Risk factor	Main findings
1	Lung cancer and occupational exposure: hospital-based case-control study	2022	Hospital-based case-control	Brey^11^	Exposure to carcinogenic agents in the hospital environment (chemical products, radiation).	Increased incidence of lung cancer among workers exposed to chemical products and radiation in hospital settings.
2	Lung cancer related to occupational exposure: an integrative review	2020	Integrative review	Brey^12^	Asbestos, welding fumes, crystalline silica, radiation in industrial sectors.	Increased incidence of lung cancer due to prolonged exposure to substances such as asbestos, welding fumes, and silica.
3	Occupational cancer illness in Brazil: an integrative literature review	2023	Integrative review	Almeida^13^	Exposure to asbestos and chemical substances in sectors such as construction and mining.	Increased cases of lung cancer in workers from sectors such as construction and mining due to exposure to carcinogenic agents.
4	The effect of occupational exposure to welding fumes on trachea, bronchus and lung cancer: A systematic review and meta-analysis	2022	Systematic review and meta-analysis	Loomis^14^	Welding fumes (heavy metals and carcinogenic compounds), particularly in the metallurgical industry.	Higher incidence of respiratory cancers among workers exposed to welding fumes in the metallurgical industry.
5	Diagnosis of asbestos-related lung diseases	2019	Diagnostic study	Harris^15^	Asbestos in sectors such as construction, shipping, and mining.	Increase in the prevalence of asbestos-related lung diseases despite stricter safety regulations.
6	Genetic damage in coal and uranium miners	2021	Genetic study	Silva Júnior^16^	Coal and uranium, causing genetic damage and increased lung cancer risk.	Higher risk of lung cancer among miners due to coal and uranium exposure, with increased genetic damage.
7	Prevention of Asbestos Exposure in Latin America within a Global Public Health Perspective	2019	Public health study	Algranti^17^	Asbestos exposure among construction and shipbuilding workers in Latin America.	Growth in asbestos exposure prevention policies, but still high exposure in specific sectors in Latin America.
8	Brazilian Thoracic Society recommendations for the diagnosis and monitoring of asbestos-exposed individuals	2024	Clinical recommendations	Santos^18^	Asbestos exposure, focusing on workers in asbestos industries.	Greater awareness of the need for continuous monitoring of asbestos-exposed workers, with a focus on early detection.
9	Sex-Specific Mortality from Asbestos-Related Diseases, Lung and Ovarian Cancer in Brazil	2022	Mortality study	Saito^19^	Asbestos, with higher prevalence of lung and ovarian cancer, with sex-based differences.	Higher mortality rate from lung cancer among men and ovarian cancer among women exposed to asbestos.
10	Scientific evidence of dockworker illness to nursing clinical reasoning	2016	Clinical study	Almeida^20^	Exposure to chemical substances in port environments, focusing on respiratory diseases.	Increase in respiratory diseases among dockworkers, with higher incidence of lung cancer due to occupational exposure.
11	Mortality from Selected Cancers among Brazilian Mechanics	2020	Mortality study	Santos^21^	Exposure to toxic substances (chemical products and heavy metals), especially among mechanics.	High mortality rate from occupational cancers among mechanics, with lung cancer being the most prominent.
12	Inflammatory and oxidative stress biomarkers in workers exposed to crystalline silica	2019	Mortality study	Scalia Carneiro^22^	Crystalline silica, with high risk of respiratory diseases and lung cancer in construction workers.	Increased incidence of lung cancer among workers exposed to crystalline silica, with evidence of severe respiratory damage.
13	Variant Enrichment Analysis in a Necropsy Series of Asbestos-Exposed Shipyard Workers	2022	Genetic study	Crovella^23^	Asbestos, focusing on shipyard workers and genetic variants associated with cancer.	Greater genetic predisposition to lung cancer among asbestos-exposed workers, based on genetic analyses.

LILACS = Latin American and Caribbean Literature on Health Sciences; SciELO = Scientific Electronic Library Online.

## Discussion

Cancer is a disease that can take a long period to manifest symptoms in the body after exposure to risk factors, making it challenging to identify the carcinogenic agent responsible for its development. This occurs because different types of cancer are associated with specific agents.^10^ In the United Kingdom, more than 8% of cancer deaths in men have been attributed to occupational exposures, with estimates indicating that over 20% of these cases were related to lung cancer. Furthermore, approximately 70% of deaths from work-related cancer occurred due to lung cancer, with more than half of these cases attributed to asbestos exposure, and the construction industry accounting for most of this exposure.^24^

In Brazil, studies investigating the relationship between occupational exposure and cancer remain limited. An analysis of theses and dissertations in the field of workers’ health, developed in graduate programs in Brazil and abroad between 1970 and 2004, identified a total of 1,018 works, of which only six (0.6%) focused specifically on cancer. This figure represents the lowest proportion among the topics covered in the research.^25^ Over the past 2 decades, this scenario has remained virtually unchanged. In contrast, a cohort study published in 2021, which analyzed mortality among former workers of an asbestos-cement plant in the city of Osasco, reported a significant increase in deaths in this population, especially from malignant neoplasms of the pleura and peritoneum, lung cancer, and cases of asbestosis.^26^

The studies analyzed confirm the strong relationship between occupational exposure and the development of lung cancer. The different methodological approaches adopted have contributed to a comprehensive understanding of the risk factors and trends associated with the disease among workers in various sectors. A study conducted by Brey et al.^11^ used a hospital-based, case-control method to investigate lung cancer incidence among workers exposed to chemical products and radiation in hospital environments, revealing a significant increase in the disease in this group. This finding is supported by another study by the same lead author, which, through an integrative review, identified asbestos, welding fumes, crystalline silica, and radiation in industrial sectors as predominant factors in the development of lung cancer.^12^

In this context, the review conducted by de Almeida et al.^13^ focuses on the Brazilian setting and highlights construction and mining as critical sectors, where exposure to carcinogenic substances continues to raise disease rates among workers. Similarly, Harris et al.^15^ analyze the impact of asbestos exposure in sectors such as shipping, mining, and especially construction, pointing out that despite regulatory advances, asbestos-related lung diseases remain a recurring problem.

The systematic review and meta-analysis conducted by Loomis et al.^14^ examined the effects of welding fume exposure, highlighting the increased incidence of respiratory cancers among workers in the metallurgical industry. The study confirms that inhalation of metallic particles and carcinogenic compounds generated during welding plays a significant role in lung carcinogenesis.

In addition, the genetic damage resulting from occupational exposure was explored by da Silva Júnior et al.^16^ and Scalia Carneiro et al.^22^, who investigated the impact of coal and uranium on miners’ health. The findings suggest that these workers face a significantly higher risk of lung cancer due to genetic alterations caused by prolonged exposure to these substances. In this context, genetic analysis of shipyard workers exposed to asbestos revealed that certain genetic variants may increase susceptibility to lung cancer, suggesting that some individuals have greater biological vulnerability to developing the disease, even under similar environmental exposure loads.^23^

Concerns regarding asbestos exposure are also addressed by Algranti et al.^17^, who discuss its presence in Latin America and emphasize the need for more effective prevention policies. This concern is shared by Santos et al.^18^, whose clinical recommendations underscore the importance of continuous monitoring for the early detection of disease in exposed workers.

The study by Saito et al.^19^ addresses sex differences in asbestos-related mortality, revealing that exposed men have a higher mortality rate from lung cancer, while exposed women show an increased incidence of ovarian cancer. Almeida & Cezar-Vaz^20^ reinforce that working conditions in port environments demonstrate that continuous exposure to chemical substances in these settings contributes to the rise in respiratory diseases such as lung cancer.

Finally, the study by Santos et al.^21^ analyzes mortality among Brazilian mechanics exposed to chemical products and heavy metals, concluding that this group presents elevated lung cancer rates.

Taken together, the evidence clearly shows that occupational exposure remains a determining factor in the development of lung cancer across various productive sectors. Although regulations and guidelines exist to mitigate these risks, the literature indicates that inadequate enforcement and insufficient monitoring continue to pose significant challenges. Therefore, measures such as strengthening the enforcement of safety standards, replacing carcinogenic substances with less harmful alternatives, and expanding early screening programs are essential to reduce the incidence of the disease among exposed workers.

## Conclusions

In summary, occupational exposure to carcinogenic substances remains a relevant factor in the development of lung cancer among workers in various sectors, particularly construction, mining, metallurgy, and health care. Although regulations have been implemented, insufficient enforcement and the lack of effective monitoring still pose significant challenges to risk mitigation. Scientific evidence highlights the urgent need for stronger prevention policies as well as early screening programs to promote the health of exposed workers. The implementation of less harmful alternatives, combined with stricter control measures, can play an essential role in reducing the incidence of lung cancer among workers.
